# TNF‐α/NF‐κB signaling epigenetically represses PSD4 transcription to promote alcohol‐related hepatocellular carcinoma progression

**DOI:** 10.1002/cam4.3832

**Published:** 2021-05-01

**Authors:** Jia’ning Shi, Shupeng Song, Shuangxing Li, Kaili Zhang, Yinghua Lan, Yongguo Li

**Affiliations:** ^1^ Department of Infectious Disease The First Affiliated Hospital of Harbin Medical University Harbin Heilongjiang China

**Keywords:** alcohol, EFA6B, HCC, p65, PSD4

## Abstract

**Background:**

Chronic alcohol consumption is more frequently associated with advanced, aggressive hepatocellular carcinoma (HCC) tumors. Alcohol adversely impacts ER/Golgi membrane trafficking and Golgi protein N‐glycosylation in hepatocytes; these effects have been attributed (in part) to dysregulated adenosine diphosphate‐ribosylation factor (ARF) GTPase signaling. Here, we investigated the role of the ARF GTPase guanine exchange factor PSD4 in HCC progression.

**Methods:**

R‐based bioinformatics analysis was performed on publicly available array data. Modulating gene expression was accomplished via lentiviral vectors. Gene expression was analyzed using quantitative real‐time PCR and immunoblotting. PSD4 promoter methylation was assessed using quantitative methylation‐specific PCR. Phospho‐p65(S276)/DNMT1 binding to the PSD4 promoter was analyzed via chromatin immunoprecipitation. We constructed ethanol/DEN‐induced and DEN only‐induced transgenic murine models of HCC.

**Results:**

We identified PSD4 as a hypermethylated, suppressed gene in alcohol‐related HCC tumors; however, PSD4 was not dysregulated in all‐cause HCC tumors. Certain HCC cell lines also displayed varying degrees of PSD4 downregulation. PSD4 overexpression or knockdown decreased and increased cell migration and invasiveness, respectively. Mechanistically, PSD4 transcription was repressed by TNF‐α‐induced phospho‐p65(S276)’s recruitment of DNA methyltransferase 1 (DNMT1), resulting in PSD4 promoter methylation. PSD4 inhibited pro‐EMT CDC42 activity, resulting in downregulation of E‐cadherin and upregulation of N‐cadherin and vimentin. Hepatocyte‐specific PSD4 overexpression reduced ethanol/DEN‐induced HCC tumor progression and EMT marker expression in vivo.

**Conclusions:**

PSD4 is a hypermethylated, suppressed gene in alcohol‐related HCC tumors that negatively modulated pro‐EMT CDC42 activity. Furthermore, we present a novel phospho‐NF‐κB p65(S276)/DNMT1‐mediated promoter methylation mechanism by which TNF‐α/NF‐κB signaling represses PSD4 transcription in HCC cells.

## Graphical abstract

PSD4 is a hypermethylated, suppressed gene in alcohol‐related HCC tumors. PSD4 decreases cell migration and invasiveness in HCC cells. PSD4 is epigenetically repressed by TNF‐α‐induced phospho‐p65(S276)’s recruitment of DNMT1.

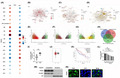

## 1 INTRODUCTION

The most common type of liver cancer is hepatocellular carcinoma (HCC), which accounts for between 80 and 90% of cases.^1^, ^2^ In 2012, there were 782,000 newly diagnosed HCC cases and 746,000 related deaths globally.^2^ Chronic alcohol consumption is recognized as a key risk factor in HCC development.^3^ Moreover, chronic alcohol consumption is more frequently associated with advanced and aggressive HCC tumors.^4^ Although alcohol is recognized as a key risk factor in HCC, its mechanism(s) of action in promoting HCC progression are currently unknown.

Alcohol alters methyl group transfer catalysis during methylation reactions, suggesting that abnormal DNA methylation may be associated with alcohol‐mediated carcinogenesis.^5^ Indeed, alcohol‐induced alterations in DNA methylation have been linked to alcohol‐related HCC.^5^ DNA methylation occurs due to DNA methyltransferases (DNMT) that conjoin a methyl group to the 5’ carbon position of the cytosine ring.^6^ Tumor suppressor genes are often silenced by DNMT‐based promoter hypermethylation; consequently, DNMT upregulation has been linked to oncogenesis.^6^ DNMT1, DNMT3A, and DNMT3B are upregulated in both alcoholic liver disease (ALD) tissue and HCC tumors.^6^, ^7^ Furthermore, elevated DNMTs are associated with a poor prognosis and often used as a predictor of survival.^6^ However, the role of DNA methylation in regulating gene expression in alcohol‐related HCC remains largely at the profiling stage.^5^, ^8^

Alcohol adversely impacts ER/Golgi membrane trafficking and Golgi protein N‐glycosylation in hepatocytes; these effects have been attributed (in part) to dysregulated adenosine diphosphate‐ribosylation factor (ARF) GTPase signaling.^9^, ^10^ ARF GTPases control key cellular processes, most notably membrane trafficking, proliferation/cell division, motility, and gene transcription.^9^, ^10^ ARF GTPase activity is negatively regulated by their bound GDP status (preventing GTP binding) by guanine nucleotide exchange factors (GEFs) and positively regulated by GTPase‐activating proteins (GAPs).^9^, ^10^ The Cancer Genome Atlas next‐generation sequencing (NGS) data reveal that ARF GTPase signaling is significantly altered in several types of cancer.^9^, ^10^ However, the role of ARF GTPases (and their associated GEFs and GAPs) in alcohol‐related HCC remains largely unexplored.

Here, using R‐based bioinformatics analysis, we identify the ARF GTPase GEF PSD4 (EFA6B) as a hypermethylated, suppressed gene in alcohol‐related HCC tumors. We also demonstrate that PSD4 functions as tumor suppressor in HCC cells via negatively modulating pro‐EMT CDC42 activity. Furthermore, we present a novel phospho‐NF‐κB p65(S276)/DNMT1‐mediated promoter methylation mechanism by which TNF‐α/NF‐κB signaling represses PSD4 transcription in HCC cells. These findings reveal that PSD4 may be a promising therapeutic target for alcohol‐related HCC.

## 2 METHODS

The Supporting Methods details the bioinformatics analysis, lentiviral constructs, qPCR, Western blotting, immunoprecipitation, immunofluorescence, cell assays, quantitative methylation‐specific PCR (qMSP), chromatin immunoprecipitation (ChIP) and Re‐ChIP assays, and the transgenic alcoholic diethylnitrosamine (DEN) murine model of HCC. Data are represented as means ± standard deviations (SDs) unless otherwise specified. SPSS was used to perform all statistical analysis. For comparison of two groups, a Student's *t* test was used. For comparison of multiple groups, a one‐way ANOVA with Bonferroni's post‐hoc testing was used. For comparison of qualitative variables, a Pearson χ^2^ or Fisher exact test was used. *p* < 0.05 was considered significant for all analyses.

## 3 RESULTS

### 3.1 Bioinformatics analysis identifies PSD4 as a key hypermethylated, repressed gene in alcohol‐related HCC

**FIGURE 1:**
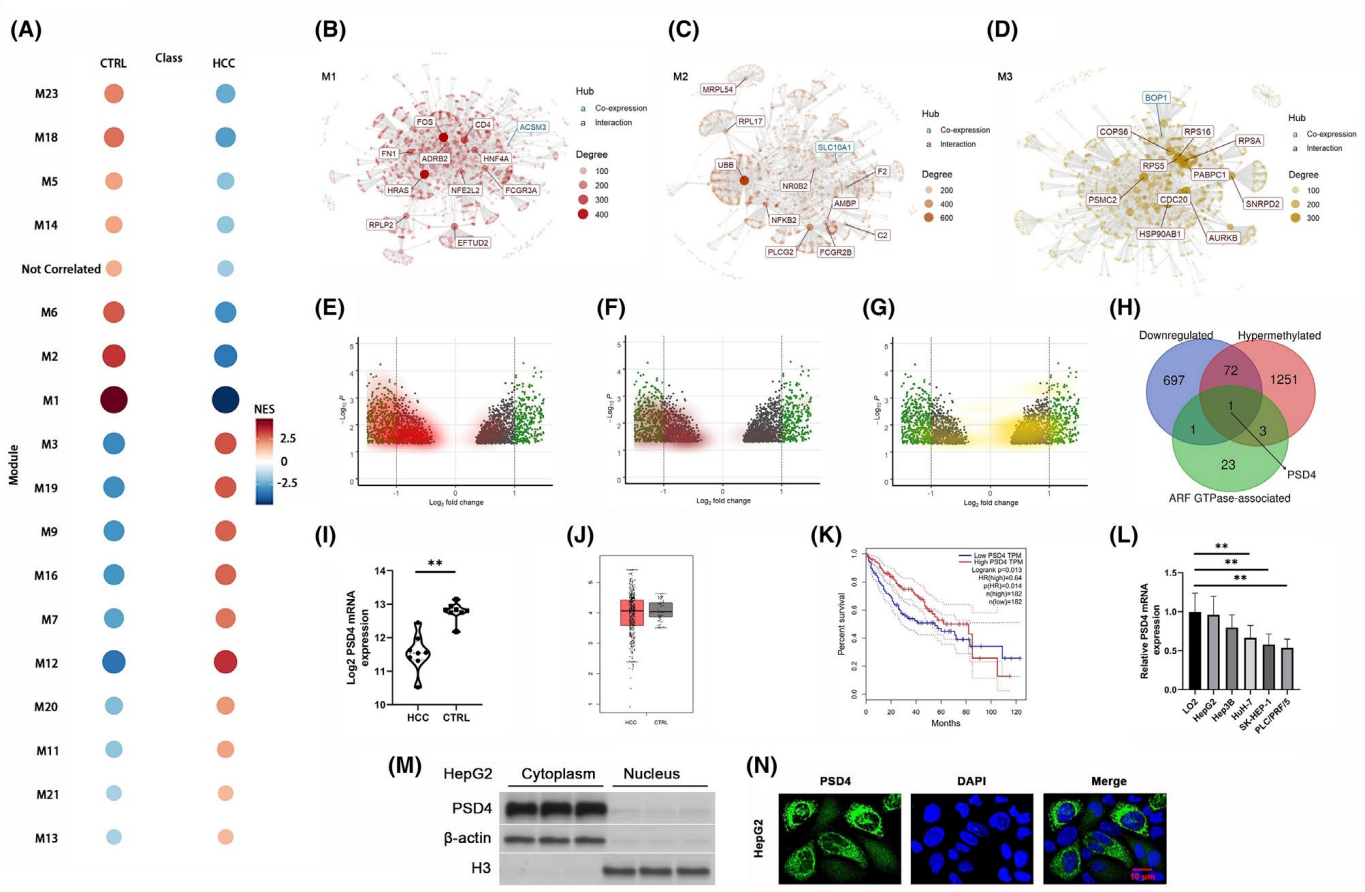
Bioinformatics identifies PSD4 as hypermethylated, suppressed gene in alcohol‐related HCC; PSD4 downregulated in HCC cell lines. (A‐D) CemiTools R analysis of gene microarray expression data (GEO accession number: GSE59261) derived from eight matched alcohol‐related HCC tumor (HCC) and normal liver control (CTRL) samples. (A) CemiTools R analysis revealed 18 distinct gene co‐expression modules differentiating HCC from CTRL. (B‐D) Network diagrams of the three highest‐ranked modules (B) M1, (C) M2, and (D) M3 depicting the size, density, and key hub genes of each module, respectively. (E‐G) Limma R‐based DEG analysis produced 2918 DEGs (adj. *p* < 0.05), 771 profoundly downregulated (log_2_FC < −1.0, left‐side green dots). Shading DEGs by module membership revealed nearly uniform suppression in M2 DEGs (panel (F), brown shading). (H) Venn analysis of profoundly downregulated M2 DEGs (log_2_FC < −1.0), hypermethylated genes (FC >1.0), and ARF GTPase‐associated genes identified PSD4 as a key candidate repressed gene in alcohol‐related HCC. (I) PSD4 mRNA expression in alcohol‐related HCC and CTRL (GSE59261 cohort). (J) PSD4 mRNA expression in all‐cause HCC (n = 369) and CTRL (n = 50) (TCGA LIUD cohort). (K) Kaplan–Meier analysis of overall survival stratified by median PSD4 expression in all‐cause HCC (n = 364) (TCGA LIUD cohort). (L) qPCR profiling of PSD4 mRNA expression in HCC cell lines and non‐malignant L02 cells. (M, N) Cytoplasmic localization of PSD4 in HepG2 cells determined by (M) subcellular fractionation and immunoblotting and (N) immunofluorescence (scale bar =10 μm). Patient data represented as medians ±IQRs and absolute ranges. Cell line data represented as means ±SDs. *p < 0.05, **p < 0.01 (one‐way ANOVA)

CemiTools R analysis of gene microarray expression data (GEO accession number: GSE59261) derived from eight matched alcohol‐related HCC tumor (HCC) and normal liver control (CTRL) samples revealed 18 distinct gene co‐expression modules differentiating HCC from CTRL (Figure 1A). We focused our analysis on the three highest‐ranked modules M1, M2, and M3 (Figure 1B‐D, Figure S1A,B). Limma R‐based DEG analysis produced 2918 DEGs (adj. *p* < 0.05), 771 (26%) of which were profoundly downregulated (log_2_FC < −1.0). Segregating DEGs by module membership revealed nearly uniform suppression in M2 DEGs (Figure 1E–G), leading us to focus on M2 DEGs. Venn analysis of the union of (i) profoundly downregulated M2 DEGs, (ii) hypermethylated genes (methylation FC >1.0), and (iii) ARF GTPase‐associated genes identified PSD4 as a key candidate repressed gene in alcohol‐related HCC (Figure 1H).

Confirming our analysis, we found PSD4 mRNA downregulation in alcohol‐related HCC tumors within the GSE59261 cohort (Figure 1I). Interestingly, PSD4 mRNA expression was not dysregulated in all‐cause HCC tumors from the TCGA LIUD cohort (Figure 1J). However, below‐median PSD4 expression was associated with worse overall survival in the TCGA LIUD cohort (Figure 1K). This evidence indicates that PSD4 is specifically downregulated in alcohol‐related HCC and may play a role in improving HCC patient survival.

### 3.2 HCC cell lines display varying degrees of PSD4 downregulation

PSD4 expression was measured in five human HCC cell lines (i.e., HepG2, Hep3B, HuH‐7, SK‐HEP‐1, and PLC/PRF/5) and the immortalized hepatocyte cell line LO2. PSD4 was downregulated in HuH‐7, SK‐HEP‐1, and PLC/PRF/5 cells when compared to L02 cells (Figure 1L). In HepG2 cells, subcellular‐fractionated immunoblotting showed that the PSD4 protein was predominantly localized in the cytoplasm (Figure 1M). Similarly, immunofluorescent staining also demonstrated cytoplasmic localization of PSD4 (Figure 1N).

### 3.3 PSD4 inhibits HCC cell proliferation, migration, and invasiveness via CDC42

**FIGURE 2:**
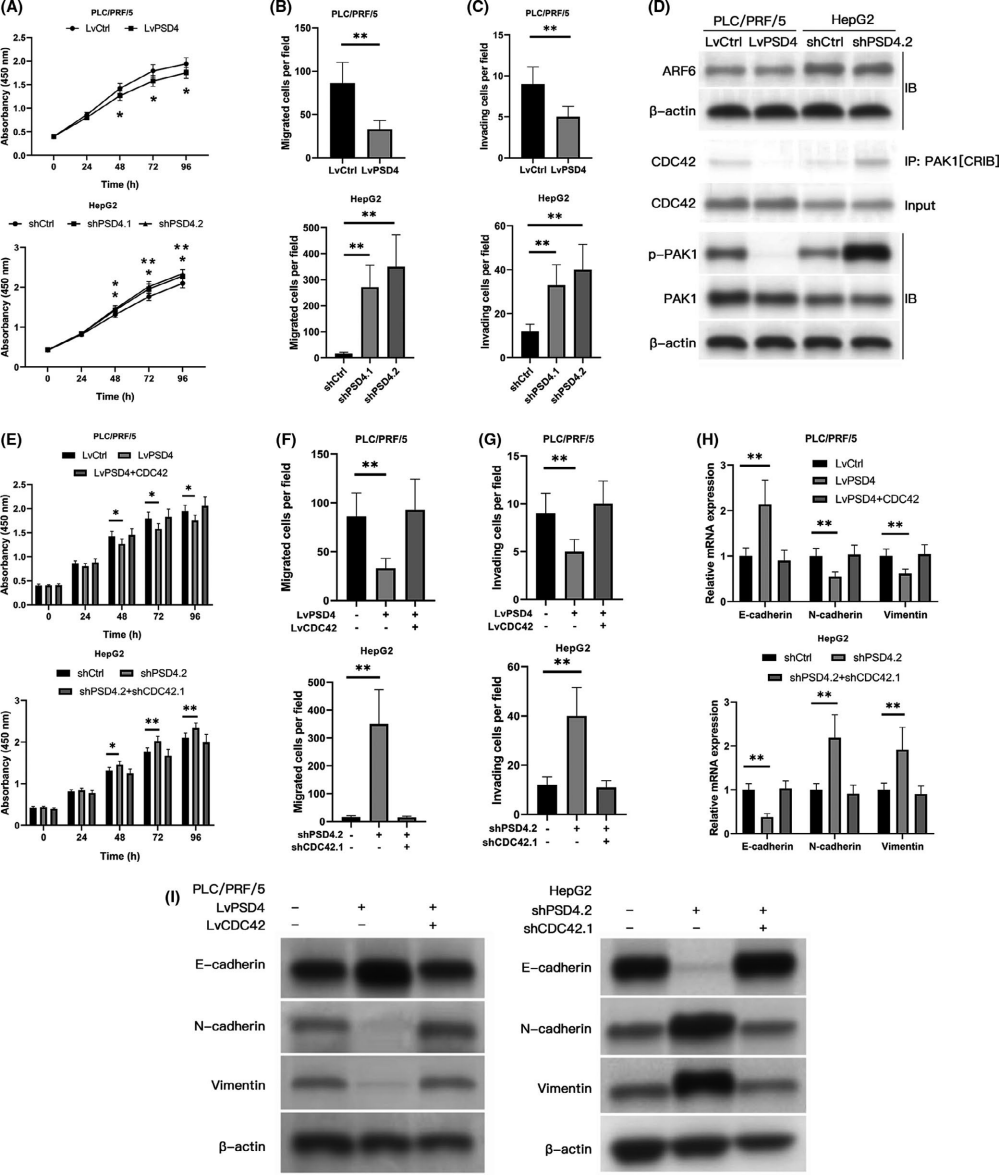
PSD4 inhibits HCC cell proliferation, migration, and invasiveness via CDC42. (A–D) We constructed stable lentiviral PSD4 overexpression in PLC/PRF/5 cells and stable PSD4 knockdown in HepG2 cells. (A) Cell proliferation determined by CCK‐8 assays. (B) Cell migration and (C) invasiveness assessed with Transwell assays (×100). (D) PSD4 overexpression inhibited CDC42/PAK1[CRIB] binding and PAK1 phosphorylation in PLC/PRF/5 cells (left), while PSD4 knockdown enhanced CDC42/PAK1[CRIB] binding and PAK1 phosphorylation in HepG2 cells (right). (E‐I) We constructed stable lentiviral PSD4 overexpression (with or without CDC42 overexpression rescue) in PLC/PRF/5 cells and stable PSD4 knockdown (with or without CDC42 knockdown rescue) in HepG2 cells. (E) Cell proliferation determined via CCK‐8 assays. (F) Cell migration and (G) invasiveness assessed with Transwell assays. (H) Gene expression and (I) protein levels of the EMT markers E‐cadherin, N‐cadherin, and vimentin. Data represented as means ± SDs. *p < 0.05, **p < 0.01 (one‐way ANOVA)

To examine the role of PSD4 in HCC tumorigenesis, we assessed the impact of PSD4 overexpression or silencing on HCC cell proliferation, migration, and invasiveness in vitro. We selected HepG2 and PLC/PRF/5 cell lines, which displayed the highest and lowest PSD4 expression, respectively, for further experiments. Based on their endogenous PSD4 expression levels, PLC/PRF/5 were transfected with a lentiviral vector (LvPSD4) to increase PSD4 expression, while HepG2 cells were transfected with one of two PSD4 shRNAs (shPSD4.1 or shPSD4.2) to decrease PSD4 expression (Figure S2A,B). LvPSD4 PLC/PRF/5 cells had decreased cell proliferation, migration, and invasiveness. In contrast, shPSD4 HepG2 cells displayed increased cell proliferation, migration, and invasiveness (Figure 2A–C). Collectively, these data demonstrate that PSD4 inhibits HCC cell proliferation, migration, and invasiveness.

PSD4 is an ARF guanine nucleotide exchange factor (GEF) of ARF6; functionally, the EFA6B‐ARF6 dyad is necessary for tight junction maintenance.^11^, ^12^ Based on a literature search for downstream targets of PSD4, we discovered Fayad et al.’s recent work describing the ARF6/CDC42/PAK1 axis as a regulatory target of PSD4 in breast cancer cells.^13^, ^14^ Consequently, we hypothesized that modulating PSD4 expression may affect ARF6/CDC42/PAK1 axis activity in HCC cells. Notably, PSD4 overexpression in PLC/PRF/5 cells did not impact ARF6 expression but reduced binding of CDC42 to PAK1’s CDC42 binding domain (PAK1[CRIB]) and PAK1 phosphorylation (Figure 2D). Conversely, PSD4 knockdown in HepG2 cells slightly decreased ARF6 protein levels but significantly enhanced CDC42/PAK1[CRIB] binding and PAK1 phosphorylation (Figure 2D). These findings confirm that PSD4 negatively regulates CDC42/PAK1 axis activity in HCC cells.

To determine whether CDC42 mediates PSD4’s inhibitory effects on HCC cell proliferation, migration, and invasiveness, PSD4 and CDC42 lentiviral vectors were transfected into PLC/PRF/5 and HepG2 cells. PSD4 and CDC42 modulation were confirmed via qPCR (Figure SC‐F). PLC/PRF/5 cells with PSD4 overexpression demonstrated reduced proliferative, migratory, and invasive capacity, which were rescued by the addition of CDC42 overexpression (Figure 2E‐G). HepG2 cells with PSD4 knockdown demonstrated enhanced proliferative, migratory, and invasive capacity, which were rescued by the addition of CDC42 knockdown (Figure 2E‐G). Consistently, PLC/PRF/5 cells with PSD4 overexpression showed enhanced E‐cadherin and decreased N‐cadherin and vimentin expression, which were rescued by the addition of CDC42 overexpression (Figure 2H,I). Conversely, HepG2 cells with PSD4 knockdown showed decreased E‐cadherin and enhanced N‐cadherin and vimentin expression (Figure 2H,I).

### 3.4 PSD4 expression suppressed by TNF‐α‐induced phospho‐p65(S276) in HCC cells

**FIGURE 3:**
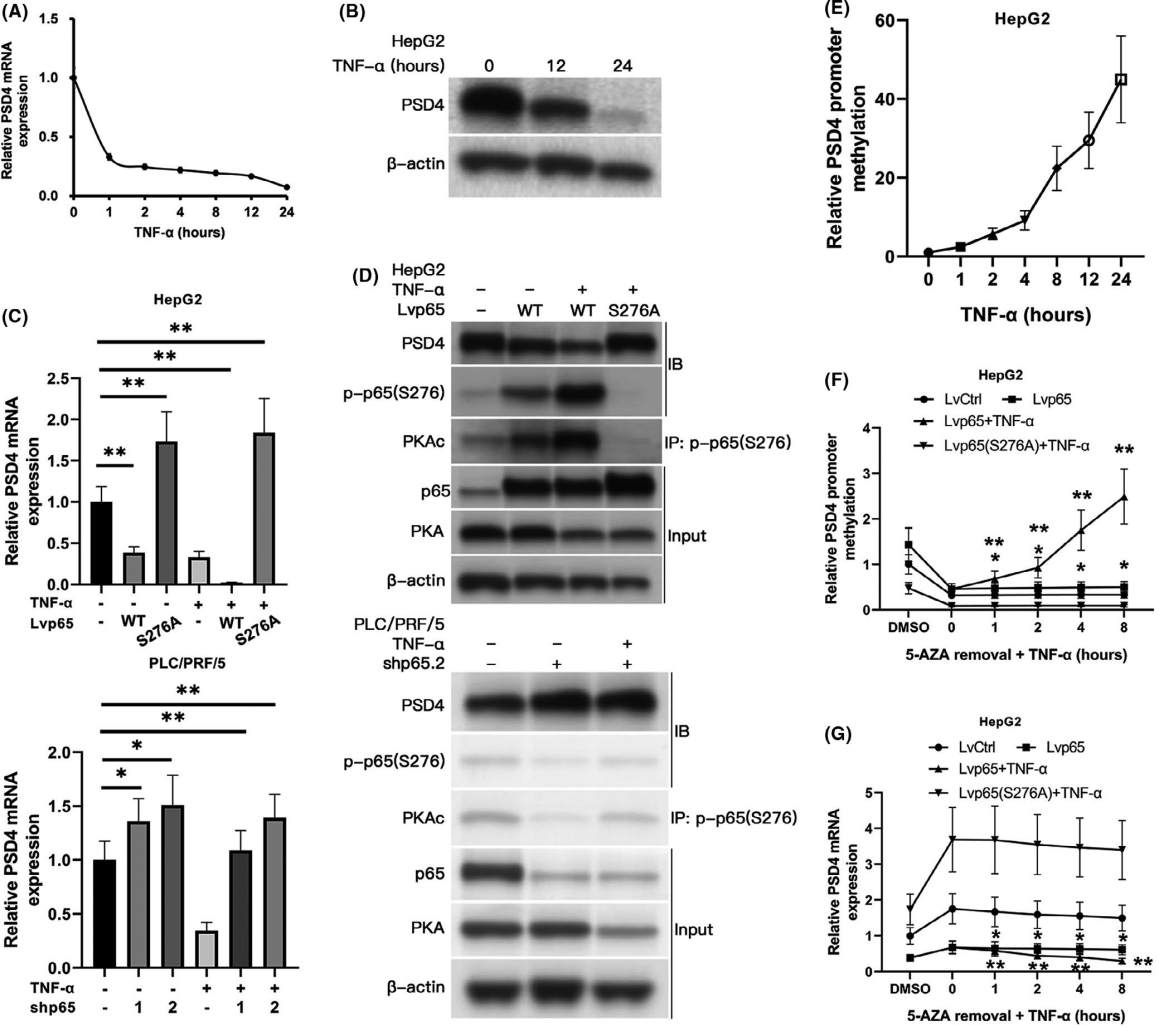
Phospho‐p65(S276) mediates TNFα‐induced PSD4 transcriptional repression via enhancing PSD4 promoter methylation. (A, B) HepG2 cells were cultured with TNFα (40 ng/ml). (A) PSD4 mRNA expression and (B) PSD4 protein expression were analyzed by qPCR and immunoblotting, respectively, at the indicated time points. (C, D) We constructed stable lentiviral WT p65 overexpression or p65 S276A overexpression in HepG2 cells (top) or stable p65 knockdown cells in PLC/PRF/5 cells (bottom). Cells were cultured under untreated or TNF‐α (40 ng/ml) for 1 hour as indicated. (C) PSD4 mRNA expression analyzed by qPCR. (D) p‐p65(S276)/PKAc binding and PSD4 protein expression analyzed by immunoblotting/immunoprecipitation. (E) HepG2 cells were cultured with TNFα (40 ng/ml). qMSP assessment of PSD4 promoter methylation. (F, G) WT p65 overexpression or p65 S276A overexpression HepG2 cells were treated with 5‐AZA (5 mM) for 5 days for global demethylation, followed by culturing with TNFα (20 ng/ml). (F) qMSP assessment of PSD4 promoter methylation. (G) Levels of PSD4 transcription analyzed by qPCR. Data represented as means ±SDs. *p < 0.05, **p < 0.01 (one‐way ANOVA)

Having uncovered CDC42 as a key downstream mediator of PSD4, we next investigated potential upstream regulators of PSD4 in HCC cells. Our CemiTool R analysis revealed that PSD4’s gene module M2 contains the NF‐κB p100 (NFKB2) as a key hub gene (Figure 1B), a key regulator of canonical and non‐canonical NF‐κB signaling.^15^, ^16^ Additionally, considering that PSD4 downregulation is unique to alcohol‐related HCC (Figure 1H,J) and ALD is associated with more pronounced canonical TNF‐α/NF‐κB signaling,^17^, ^18^ we hypothesized that enhanced TNF‐α/NF‐κB signaling may regulate PSD4 expression in HCC cells. Accordingly, we observed that TNF‐α reduced both mRNA and protein levels of PSD4 in a time‐dependent manner in HepG2 cells (Figure 3A,B).

Previous studies have shown that a specific phosphorylated form of the NF‐κB p65 subunit—phospho‐p65(S276)—functions as a transcriptional repressor downstream of TNF‐α/cAMP‐dependent protein kinase (PKA) signaling in cancer cells.^19^, ^20^ Notably, our ConTra v3 analysis identified a p65/RELA binding site on the PSD4 promoter located approximately 2.5 kbp upstream of the transcription start site (TSS) (Figure S3A–C), leading us to hypothesize that TNF‐α represses PSD4 transcription via p‐p65(S276). In HepG2 cells, overexpression of WT p65 (but not the S276A mutant) led to PSD4 mRNA downregulation under TNF‐α conditions (Figure 3C). Conversely, in PLC/PRF/5 cells, lentiviral p65 knockdown resulted in PSD4 mRNA upregulation under TNF‐α conditions (Figure 3C). Furthermore, overexpression of WT p65 (but not the S276A mutant) in HepG2 cells stimulated PKA catalytical subunit (PKAc)‐p‐p65(S276) binding and PSD4 protein downregulation under TNF‐α conditions (Figure 3D). Conversely, in PLC/PRF/5 cells, lentiviral p65 knockdown decreased PKAc‐p‐p65(S276) binding and enhanced PSD4 protein expression under TNF‐α conditions (Figure 3D).

### 3.5 P‐p65(S276) mediates TNF‐α‐induced PSD4 promoter methylation and transcriptional repression

PSD4 is hypermethylated and repressed in alcohol‐related HCC tumors.^5^ As promoter methylation is frequently associated with gene repression,^21^ we hypothesized that TNF‐α’s suppression of PSD4 transcription may be mediated through enhanced PSD4 promoter methylation. Indeed, we found two CpG islands on the PSD4 promoter localized near the p65/RELA binding site (approx. 2.5 kbp upstream of the TSS) (Figure S4). *In vitro*, TNF‐α increased PSD4 methylation in a time‐dependent manner in HepG2 cells (Figure 3E). To examine the role of p‐p65(S276) in TNF‐α induced PSD4 promoter methylation, WT p65 and S276A mutant HepG2 cells were exposed to 5’‐aza‐2’‐deoxycytidine (5‐AZA) to induce global hypomethylation. Following removal of 5‐AZA, the PSD4 promoter was remethylated in TNF‐α‐treated WT p65 cells (Figure 3F). However, in S276A mutant cells, the PSD4 promoter remained hypomethylated despite TNF‐α exposure (Figure 3F). qPCR demonstrated that this methylation was negatively correlated with PSD4 mRNA levels in the TNF‐α exposed WT p65 cells (Figure 3G). Under non‐TNF‐α‐treated conditions, p65 expression produced a marginal impact on methylation or transcription of PSD4 (Figure 3F,G). This combined evidence suggests that p‐p65(S276) is critically involved in TNF‐α‐mediated PSD4 promoter methylation and transcriptional repression.

### 3.6 p65’s recruitment of DNMT1 to the PSD4 promoter enhances repressive PSD4 promoter methylation

**FIGURE 4:**
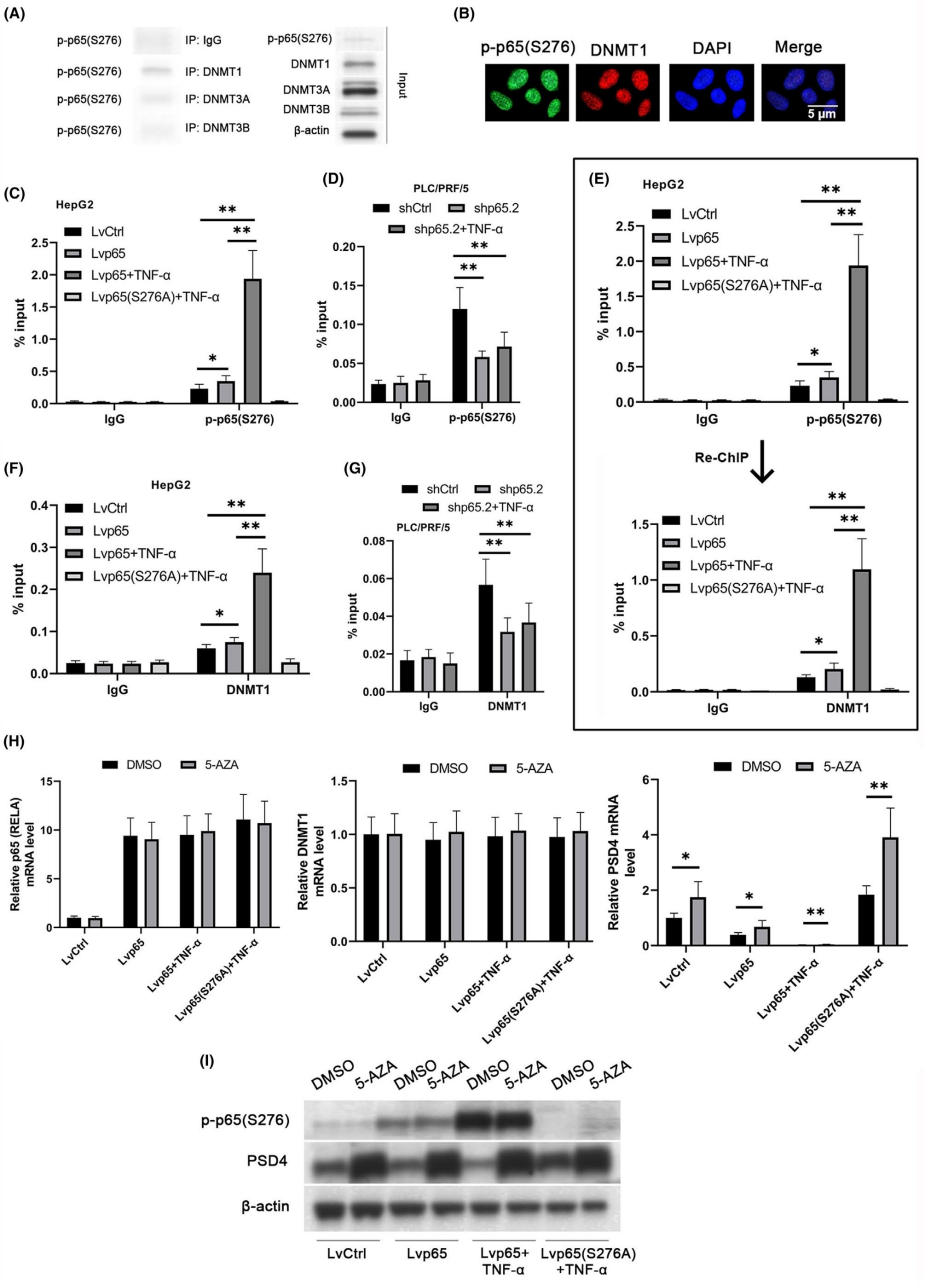
Phospho‐p65(S276)’s recruitment of DNMT1 enhances PSD4 promoter methylation and transcriptional repression. (A) Immunoprecipitation in PLC/PRF/5 cells showing p‐p65(S276)’s interaction with DNMT1, not DNMT3A or DNMT3B. (B) Immunofluorescent nuclear co‐localization of p‐p65(S276) and DNMT1 in PLC/PRF/5 cells (scale bar =5 μm). (C‐G) We constructed stable lentiviral WT p65 overexpression or p65 S276A overexpression in HepG2 cells or stable p65 knockdown cells in PLC/PRF/5 cells. ChIP assays were performed under untreated or TNF‐α conditions as indicated. (C, D) ChIP assays showing p‐p65(S276) binding to the PSD4 promoter in (C) HepG2 cells and (D) PLC/PRF/5 cells. (E) ChIP‐Re‐ChIP assays confirming p‐p65(S276)‐DNMT1 binding to the PSD4 promoter in HepG2 cells. (F, G) ChIP assays showing DNMT1 binding to the PSD4 promoter in (F) HepG2 cells and (G) PLC/PRF/5 cells. (H‐J) The aforementioned HepG2 cell lines were cultured for 48 hours under DMSO‐ or 5‐AZA‐treated conditions. (H) Gene expression of p65 (RELA), DNMT1, and PSD4 determined via qPCR. (I) p‐p65(S276), DNMT1, and PSD4 protein levels determined via Western blotting. Data represented as means ± SDs. *p < 0.05, **p < 0.01 (one‐way ANOVA)

We next examined the underlying mechanism of p‐p65(S276)‐mediated methylation of the PSD4 promoter. By immunoprecipitation, we explored the interaction between p‐p65(S276) and the three major DNA methyltransferases in HCC: DNMT1, DNMT3A, and DNMT3B.^22^ Notably, p‐p65(S276) interacted with DNMT1 (but not with DNMT3A or DNMT3B) (Figure 4A). This p‐p65(S276)‐DNMT1 interaction was validated through immunofluorescent co‐localization visible in the nuclei (Figure 4B).

The ENCODE ChIP‐seq database has identified the PSD4 promoter as a target of p65 (RELA),^23^ which we confirmed through our ChIP assays. Briefly, p‐p65(S276) was enriched at approx. 2.5 kbp upstream of the PSD4 TSS; this signal was enhanced by overexpression of WT p65 (but not the S276A mutant) in HepG2 cells and reduced by p65 knockdown in PLC/PRF/5 cells under TNF‐α conditions (Figure 4C,D). Furthermore, ChIP/Re‐ChIP assays highlighted co‐occupancy of p‐p65(S276) and DNMT1 at this PSD4 promoter region under TNF‐α conditions (Figure 4E). We also observed an increase in DNMT1 binding by overexpression of WT p65 (but not the S276A mutant) in HepG2 cells, and a decrease in DNMT1 binding by p65 knockdown in PLC/PRF/5 cells under TNF‐α conditions (Figure 4F,G). This combined evidence suggests that TNF‐α‐induced p‐p65(S276) recruits DNMT1 to the PSD4 promoter, which enhances DNA methylation and PSD4 transcriptional repression.

We next explored whether DNMT1 inhibition by 5‐AZA could restore PSD4 expression in WT p65‐overexpressing HepG2 cells and, consequently, the metastatic phenotype. 5‐AZA decreased DNMT1 protein levels but increased PSD4 gene and protein levels in WT p65‐overexpressing HepG2 cells under TNF‐α conditions (Figure 4H,I). Our data demonstrate that PSD4 downregulation in WT p65‐overexpressing HCC cells is dependent upon DNMT1 activity.

### 3.7 Construction and characterization of hepatocyte‐specific PSD4 overexpression transgenic mice

To investigate the effects of PSD4 *in vivo*, we constructed hepatocyte‐specific Psd4‐overexpressing transgenic mice by placing the murine *Psd4* cDNA under the control of the hepatocyte‐specific murine albumin (Alb) promoter. This Alb‐PSD4 transgene construct (Figure S5A) was injected into zygotic pronuclei isolated from E0.5‐day pregnant female mice. After brief culturing, these transgenic zygotes were transferred into pseudo‐pregnant females. Following birth, tail clippings from transgenic Alb‐PSD4 (TG^Alb‐PSD4^) and non‐transgenic (non‐TG) neonate littermates were screened for the Alb‐PSD4 transgene by qPCR (Figure S5B). To validate hepatocyte‐specific expression of M2‐FLAG‐PSD4 in TG^Alb‐PSD4^ mice, hepatocytes and non‐heptocyte Kupffer cells were isolated from liver tissue samples. Immunoblotting of each cell fraction with an anti‐M2‐FLAG antibody confirmed that the Alb‐PSD4 transgene construct was solely expressed in TG^Alb‐PSD4^ hepatocytes (Figure S5C).

Following birth, mice were maintained on a regular chow diet to measure growth rates, liver/body weight ratios, and heart/body weight ratios. The differences in growth rates were not statistically significant, and TG^Alb‐PSD4^ mice and non‐TG mice achieved statistically similar adult weights for males and females (Figure S5D). There was also no differences in the liver/body weight ratios or heart/body weight ratios of TG^Alb‐PSD4^ mice in comparison to non‐TG littermates at 4 months (Figure S5E,F).

### 3.8 In vivo alcohol‐induced HCC tumorigenesis and EMT markers suppressed by hepatocyte‐specific PSD4 overexpression

**FIGURE 5:**
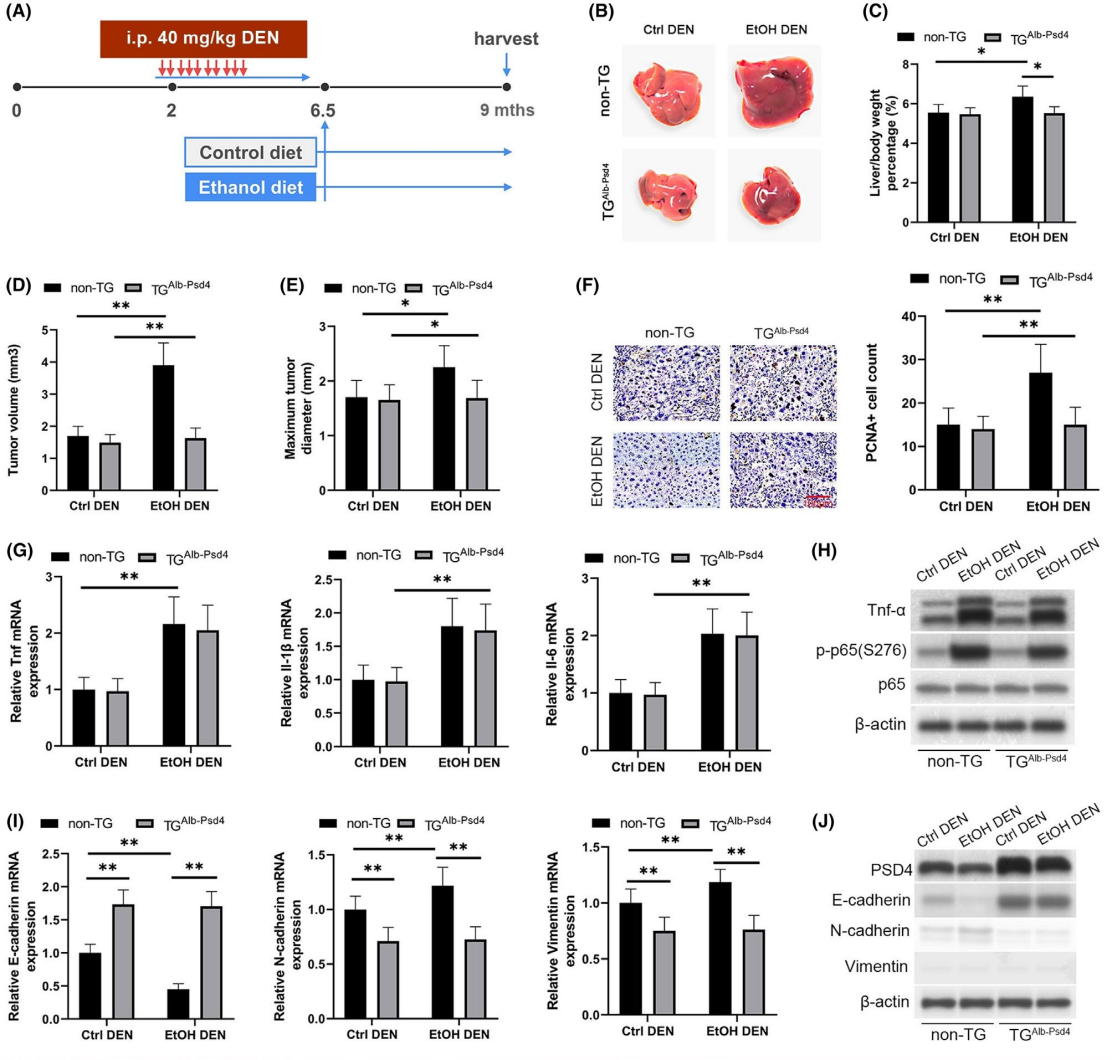
Ethanol/DEN‐induced HCC tumorigenesis and EMT markers suppressed by hepatocyte‐specific PSD4 overexpression. (A) Schematic of the ethanol/DEN‐induced murine model of HCC. Four mouse cohorts were randomly constructed by i.p. DEN injection (40 mg/kg body weight) (n = 6 male mice per cohort): DEN non‐TG, EtOH/DEN non‐TG, DEN TG^Alb‐PSD4^, and EtOH/DEN TG^Alb‐PSD4^. Mice were weighed and euthanized 7 months after DEN treatment. (B) Representative images displaying HCC tumor development in the four cohorts. (C) Liver/body weight ratios in the four cohorts. (D) Tumor volumes and (E) maximum tumor diameters in the four cohorts. (F, G) Cell proliferation determined by PCNA staining in the four cohorts. (F) Representative staining images (scale bar, 100 μm) and quantification of PCNA+cells per field. (G) Gene expression of the inflammatory markers Tnf, Il‐1β, and Il‐6 in the four cohorts determined via qPCR. (H) Protein levels of Tnfα, p‐p65(S276), and p65 via immunoblotting. (I) Gene expression of the EMT markers E‐cad, N‐cad, and Vim in the four cohorts determined via qPCR. (J) Protein levels of PSD4 and the EMT markers E‐cadherin, N‐cadherin, and vimentin determined via immunoblotting. Data represented as medians ±IQRs and absolute ranges. *p < 0.05, **p < 0.01 (one‐way ANOVA)

To investigate whether hepatocyte‐specific PSD4 overexpression has an impact on alcohol‐induced HCC tumor progression, we utilized a previously reported murine model of ethanol/DEN‐induced HCC (Figure 5A). By gross observation, ethanol/DEN non‐TG mice displayed larger liver tumor masses when compared to DEN non‐TG mice (Figure 5B), an effect reduced in TG^Alb‐PSD4^ mice. Furthermore, ethanol/DEN non‐TG mice displayed dramatic increases in liver/body weight ratios, tumor volumes, maximum tumor diameters, and PCNA+cell counts relative to DEN non‐TG mice (Figure 5C‐F), effects reduced in TG^Alb‐PSD4^ mice. Similar to human ALD,^24^ the NF‐κB‐associated inflammatory markers Tnf, IL‐1β, and Il‐6 were elevated in the livers of ethanol/DEN mice relative to DEN mice, a factor unaffected by TG^Alb‐PSD4^ status (Figure 5G). We confirmed Tnf‐α/p‐p65(S276) axis upregulation in the livers of ethanol/DEN mice relative to DEN mice, a factor unaffected by TG^Alb‐PSD4^ status (Figure 5H). mRNA and protein levels of EMT genes E‐cadherin, N‐cadherin, and vimentin were dysregulated in the livers of ethanol/DEN non‐TG mice relative to DEN non‐TG mice (Figure 5I,J), effects rescued in TG^Alb‐PSD4^ mice. These data demonstrate that hepatocyte‐specific PSD4 overexpression reduces alcohol‐induced HCC tumorigenesis and EMT marker expression in mice.

## 4 DISCUSSION

Here, we demonstrate that PSD4 is downregulated in alcohol‐related HCC tumors and certain HCC cell lines. Moreover, below‐median PSD4 expression in HCC tumors is associated with inferior survival outcomes. Functionally, PSD4 inhibited HCC cells proliferation, migration, and invasiveness in vitro and suppressed alcohol‐induced HCC tumor progression *in vivo*. At the molecular level, PSD4 transcription is epigenetic silenced via enhanced PSD4 promoter methylation by TNF‐α‐induced p‐p65(S276)/DNMT1 complex binding.

TNF‐α promotes the migratory and invasive capabilities of HCC cells.^25^ Furthermore, TNF‐α induces EMT in HCC cells by downregulating the epithelial marker E‐cadherin (CDH1) but upregulating the mesenchymal markers N‐cadherin and vimentin.^25^ As observed here, TNF‐α‐induced p‐p65(S276) has previously been implicated in methylation‐associated transcriptional repression of tumor suppressor genes. For example, enhanced TNF‐α signaling induces methylation‐associated transcriptional repression of the tumor suppressor BRMS1 via promoting p‐p65(S276)/DNMT1 complex formation in NSCLC cells. Moreover, p‐p65(S276) recruits DNMT1 to methylate the CRMP4 promoter and transcriptional repress CRMP4 in prostate cancer cells.^26^ Therefore, p‐p65(S276)/DNMT1‐mediated transcriptional repression appears to be a key theme across various cancer types and deserves further investigation.

Other than the ARF GTPase ARF6, little is known about the downstream targets of PSD4.^11^ Fayad et al.’s recent work describes the ARF6/CDC42/PAK1 axis as a regulatory target of PSD4 in breast cancer cells.^13^, ^14^ Notably, we found that PSD4 did not impact ARF6 expression but did inhibit CDC42‐PAK1[CRIB] binding and PAK1 phosphorylation, indicating suppression of CDC42/PAK1 signaling. CDC42 is a Rho GTPase that plays a key oncogenic role via regulating cytoskeletal dynamics, cell proliferation, EMT, cell migration, and invasiveness.^27^ CDC42’s effector PAK1 promotes Akt/β‐catenin activity in HCC cells, thereby promoting HCC cell proliferation.^28^ Additionally, PAK1 upregulates the EMT inducer Snail in HCC cells, promoting HCC cell migration and invasiveness.^28^ Accordingly, recent evidence now supports that CDC42/PAK1 activity downregulates E‐cadherin expression and upregulates N‐cadherin and vimentin expression in HCC cells.^29^ This cadherin switching—E‐cadherin downregulation coupled with N‐cadherin upregulation—is a key EMT indicator.^30^ Moreover, vimentin upregulation is also associated with EMT.^31^ Here, we found that PSD4 reduced cadherin switching and vimentin expression in HCC cells in a CDC42‐dependent manner, indicating PSD4’s suppression of EMT via CDC42.

A number of *in vivo* HCC mouse models are based on intraperitoneal injection of diethylnitrosamine (DEN), a carcinogenic DNA alkylator.^32^ Here, consistent with previous work by Brandon‐Warner et al., Ambade et al., and Yan et al.,^33^, ^34^, ^35^ the addition of a chronically administered ethanol liquid diet promotes liver inflammatory markers and HCC tumor progression in DEN‐exposed male mice. This murine model displays face validity, as alcoholic steatohepatitis potentiates liver inflammation and HCC risk in humans.^36^ Consistent with our in vitro findings, PSD4 suppressed ethanol/DEN‐induced HCC progression along with reducing cadherin switching and vimentin expression in HCC tumor cells in vivo. Given that PSD4 downregulation is specific to alcohol‐related HCC, our evidence suggests that targeting PSD4 may be especially effective in HCC patients with a history of chronic alcohol use.

In conclusion, this study identifies PSD4 as a hypermethylated, suppressed gene in alcohol‐related HCC tumors. We also demonstrate that PSD4 functions as tumor suppressor in HCC cells via negatively modulating pro‐EMT CDC42 activity. Furthermore, we present a novel p‐p65(S276)/DNMT1‐mediated promoter methylation mechanism by which TNF‐α/NF‐κB signaling represses PSD4 transcription in HCC cells. These findings reveal that PSD4 may be promising therapeutic target for alcohol‐related HCC.

## CONFLICTS OF INTEREST

None.

## AUTHORS’ CONTRIBUTIONS

Conceived and designed the study: SJN. Performed the experimental procedures: SJN, SSP, LSX, and ZKL. Analyzed the data: ZKL and LYH. Drafted the manuscript: LYG.

## DECLARATION

All protocols and procedures were approved by the Ethics Committee of the First Affiliated Hospital of Harbin Medical University. The established HCC cell lines HepG2 (HB‐8065), Hep3B, HuH‐7, SK‐HEP‐1, and PLC/PRF/5 and the hepatocyte cell line LO2 were obtained from American Type Culture Collection (ATCC) in October–November 2019. All cell lines were commercially authenticated by short‐tandem repeats (STR) genotyping in November 2019 by Guangzhou Jenniobio Co., Ltd. (Guangzhou, China).

## Supporting information

Supplementary Material

## Data Availability

The data underlying this article will be shared on reasonable request to the corresponding author.
