# Supplementary material for: TNF‐α/NF‐κB signaling epigenetically represses PSD4 transcription to promote alcohol‐related hepatocellular carcinoma progression
Source: Cancer Med. 2021 May 1;10(10):3346–57. doi: 10.1002/cam4.3832 (PMC8124102; doi:10.1002/cam4.3832)
Supplement: Supplementary file 1 — Supplementary Material [file CAM4-10-3346-s001.docx]

**SUPPLEMENTARY INFORMATION FOR**

**TNF-α/NF-κB signaling epigenetically represses PSD4 transcription to promote alcohol-related hepatocellular carcinoma progression**

**SUPPLEMENTARY METHODS**

**Bioinformatics analysis of alcohol-related HCC gene expression data**

Bioinformatics analysis was performed on previously-published Gene Expression Omnibus (GEO) data derived from alcohol-related HCC tumors and matching normal liver tissue surgically resected from eight adult male HCC patients (60-82 years) with at least three units/day alcohol intake × twenty years and negative viral serology [^1^](#_ENREF_1). The NimbleGen Homo sapiens HG18 expression array data was accessed from the GEO repository (accession number: GSE59261).

The CEMiTool R package was used to identify gene co-expression gene modules associated with the alcohol-related HCC phenotype using the raw GSE59261 expression array data [^2^](#_ENREF_2). The data was processed with the CEMiTool package using CEMiTool’s default parameters (i.e., variance-based gene filtering (*P*<0.1), β=14, Pearson correlation, dissimilarity threshold for hierarchical clustering=0.8, minimum genes/module=30, determination coefficient=0.8). CEMiTool overrepresentation analysis for the identified gene modules was performed with the Human Reactome annotations.

The GSE59261 expression array data were then normalized, log-transformed, and filtered in R for differentially-expressed gene (DEG) analysis. The Limma R package was used to identify DEGs between alcohol-related HCC tumors and normal liver tissue with a moderated *t*-test using a Benjamini & Hochberg adjusted *P-*value threshold < 0.05 for multiple testing.

**Cell lines**

The established HCC cell lines HepG2, Hep3B, HuH-7, SK-HEP-1, and PLC/PRF/5 and the hepatocyte cell line LO2 were obtained from American Type Culture Collection (ATCC). All cell lines were cultured in a 5% CO_2_ humidified atmosphere at 37°C in Dulbecco’s Modified Eagle Medium (DMEM) supplemented with 10% FBA, 1% penicillin/streptomycin, and high glucose. Media was replaced every 24 hours.

**Lentiviral constructs**

For gene overexpression, lentiviral particles containing cDNAs of human *PSD4* (NM_012455.2), *p65/RELA* (NM_021975), *CDC42* (NM_001039802.1), and negative control Renilla luciferase (*Rluc*) were purchased from Genechem (China). For gene knockdown, lentiviral particles containing shRNA oligonucleotide sequences against human PSD4 (sc-94601-V), p65/RELA (sc-29410-V), CDC42 (sc-29256-V), and negative control shRNA (sc-108080) were obtained from Santa Cruz Biotechnology. Cells were incubated for 8 hours in six-well plates with viral supernatants (1 ml) and polybrene (5 μg/ml). Puromycin was used to select positively-transfected cells.

**Cell proliferation, migration, and invasiveness assays**

Cell Counting Kit-8 (CCK-8) was used to determine cell proliferation. In brief, cells were plated in 96-well plates then cultured for a total of 96 hours with measurements taken every 24 hours. Assays were ran on a microplate reader (450 nm) following a 2-hour incubation at 37°C.

Cell migration and invasiveness were measured using 24-well Transwell assays. In brief, the 8 μm-pore Transwell filter without or with Matrigel (migration and invasiveness, respectively) before a 200-μl single cell suspension of 2-5 × 10^4^ cells per well in serum-free media was added to the top chamber. In the bottom chamber, 600 μl of media supplemented with FBS (10%) and TGF‐β (10 ng/ml) was added. After a 48-72 hour incubation at 37°C, any remaining cells in the top chamber were removed. Each chamber was then paraformaldehyde (4%)-fixed and labelled with crystal violet (0.1%). Cell that migrated to the bottom chamber were photographed and quantifed from 5 fields selected at random using an inverted microscope (x100).

**Quantitative real-time PCR (qPCR)**

TrIzol reagent (Invitrogen) was used to extract total RNA. PrimeScript reagent (Takara Bio) was used to generate cDNA, and SYBR Green PCR mix (Takara) was used for PCR amplification and detection. qPCR was run on the 7500 RT-PCR system (Applied Biosystems). All qPCR primers were purchased from Origene; the primer sequences are published on the Origene website (<https://www.origene.com>). Relative gene expression was normalized to the housekeeping control β-actin and calculated using the 2^−ΔΔCt^ method.

**Western blotting**

Where stated, cytosolic and nuclear fractions were separated using the PARIS Isolation Kit (Life Technologies) prior to immunoblotting. Protein lysates were obtained through the use of RIPA buffer supplemented with PMSF on ice. A five-minute centrifugation (x 14,000 g) at 4 ℃ was used to pellet debris, and cell supernatants were collected. BCA protein assays were used to determine protein concentration. Lysates were then subjected to SDS-PAGE separation before being heat transferred onto a PVDF membrane. Membranes were blocked using non-fat milk (5%) in TBS-Tween for one hour before being incubated overnight with the following primary antibodies at 4°C: PSD4 (PA5-31837, Invitrogen), CDC42 (sc-8401, SCBT), ARF6 (sc-7971, SCBT), phospho-PAK1(Thr423) (#2601, CST), PAK1 (#2602, CST), p-p65(S276) (ab106129, Abcam), p65 (ab32536, Abcam), DNMT1 (sc-271729, SCBT), DNMT3A (sc-373905, SCBT), DNMT3B (sc-81252, SCBT), M2-FLAG (#14793, CST), E-cadherin (ab76055, Abcam), N-cadherin (ab18203, Abcam), vimentin (ab137321, Abcam), histone H3 (ab1791, Abcam), and β-actin (ab8227, Abcam). The species-appropriate HRP-conjugated secondary antibodies were obtained from Abcam. Protein bands were detected using ECL.

**Immunoprecipitation**

Cells were lysed with Tris-buffered saline (pH 7.4) before being incubated with the aforedescribed primary antibodies against IgG, PAK1[CRIB], p-p65(S276), DNMT1, DNMT3A, or DNMT3B at 4°C with rotation overnight. Next, protein beads (A/G) were added to the lysates and incubated for a further 4-6 hours. Centrifugation (500 g at 4°C for 2 minutes) and three TBS washes were used to isolate the beads. Finally, the beads were eluted with loading buffer, and samples were analysed by Western blotting.

**Immunofluorescence**

Cells were plated onto glass slides and washed with PBS three time before being methanol fixed at 30°C for 15 minutes. Next, a 1-hour incubation at 37°C with BSA (5%) was used to block the samples before an overnight incubated at 4°C with the aforedescribed primary antibodies against PSD4, p-p65(S276), or DNMT1. The next day, slides were washed three times with PBS before a 1.5-hour incubation at room temperature with AF488-conjugated secondary antibody. Nuclei were counter stained with DAPI or Hoechst as indicated. Fluorescence was assessed using a confocal laser scanning microscope (Zeiss).

**Quantitative methylation-specific PCR (qMSP)**

qMSP was performed as previously described [^3^](#_ENREF_3). Briefly, genomic DNA was isolated using the DNeasy Blood and Tissue Kit (Qiagen) and then sodium bisulphite-modified with the EZ DNA Methylation Kit (Zymo Research). Bisulphite-modified DNA was used as a PCR template with primers designed with the online MethPrimer v2 tool (http://www.urogene.org/methprimer2) specific to the upstream PSD4 promoter region containing the CpG islands and the p65/RELA binding site (−3000 to -2000 from the PSD4 TSS). qMSP was run on a 7500 RT-PCR system (Applied Biosystems) with SYBR Green PCR mix (Takara). GAPDH served as the housekeeping control.

**Chromatin immunoprecipitation (ChIP) and Re-CHIP assays**

ChIP and Re-CHIP assays were performed using a standard protocol as previously described [^4^](#_ENREF_4).

**Mice housing and care**

WT C57BL/6J and DBA/-2J mice (The Jackson Laboratory) and genetically-modified mice were bred under standard conditions. Animals were maintained on a natural day-night cycle with a regular mouse chow diet (China National Standard GB14924.3-2010, Xi’an Fengwei Animals, Ltd., Xi’an, China). All animals were euthanized by cervical dislocation.

**Construction of hepatocyte-specific Psd4 overexpression transgenic mice**

Hepatocyte-specific Psd4 overexpression transgenic mice were constructed and characterized accordingly a previously described protocol [^5^](#_ENREF_5) with minor modifications . Briefly, total RNA was extracted from mouse liver with TrIzol reagent (Invitrogen) and separated by 0.7 % agarose electrophoresis for 28S RNA, 18S RNA, and 5.8S RNA. qPCR was used to amplify the mouse *Psd4* cDNA sequence (NM_177611.3). Poly(A)-RNA from the total RNA pool was employed to generate the first-strand cDNA with a cDNA synthesis kit (Gibco BRL).

To distinguish transgenic overexpression of the Psd4 protein, a M2-FLAG sequence (Asp-Tyr-Lys-Asp-Asp-Asp-Asp-Lys) was inserted before the *Psd4* cDNA within the expression construct. The M2-FLAG::*Psd4* fusion cDNA construct was subcloned under the control of a murine albumin (*Alb*) promotor to ensure hepatocyte-specific expression. Briefly, the murine *Alb* promotor, SV40 early region transcription terminator, and poly(A) site were placed within the 3′-UTR. We employed Not I and Sal I restriction sites on the 5′ end of M2-FLAG and 3′ end of Psd4 cDNA, respectively, to insert the M2-FLAG::*Psd4* fusion cDNA construct into the Alb-SV40-polyA backbone. The CsCl-purified transgene construct was then prepared for pronuclei injection and was microinjected into C57BL/6J zygotes, after which the injected zygotes were implanted into the oviducts of pseudopregnant DBA/-2J females. The transgenic mice were maintained in the DBA/-2J inbred strain.

**Characterization of hepatocyte-specific Psd4 overexpression transgenic mice**

Following birth, mouse hepatocytes and non-hepatocyte Kupffer cells were isolated from mouse liver tissue as previously described [^6^](#_ENREF_6). Total RNA was extracted from each cell fraction with TrIzol reagent (Invitrogen), and qPCR was performed as described above. Protein samples extracted from each cell fraction were separated on a 15% Tris-glycine gel (Life Technology), and Western blotting was performed with a primary anti-M2-FLAG antibody mentioned above. For Northern blotting analyses, RNA extracted from each cell fraction were run on a 4%-formaldehyde agarose gel with formalin. RNA was transferred onto a nitrocellulose membrane, which was then hybridized with H3-CTP-labeled M2-FLAG cDNA probe.

**Construction of the ethanol/DEN murine model of HCC**

Two-month-old transgenic (TG^Alb-Psd4^) mice and non-transgenic (non-TG) littermates were weighed and subsequently injected intraperitoneally (i.p.) with DEN (40 mg/kg body weight, Sigma) a total of 10 times with 4-day intervals. Mice were randomly separated into four groups by random number generation for the following 2.5-month regimens: TG^Alb-Psd4^ mice fed a control diet, non-TG mice fed a control diet, TG^Alb-Psd4^ mice fed a liquid ethanol (5%) diet (TROPHIC, China), and non-TG mice fed a liquid ethanol (5%) diet.

After 7 months, mice were weighed and scarified. Liver were isolated, weighed and investigated for any visible lesions. Liver tissues was then taken from the sample lobe and snap frozen for further analysis. For H&E, liver tissues were fixed using acidic buffer formalin (12%), paraffin-embedded and sliced into section (6 µm). Next, samples were deparaffinized, dehydrated and H&E stained. For IHC staining, liver tissues were fixed using neutral buffered formalin (12%). An overnight incubation with anti-PCNA (ZSGB-Bio, China) followed by incubation with an HRP-conjugated secondary antibody (ZSGB-Bio, China). The 3,3-diaminobenzidine kit (Vector Labs) was used to develop staining. All tissues were analyzed using light microscopy.

**SUPPLEMENTARY FIGURES**

**Fig. S1. Additional results from the CemiTools analysis**

**(A)** Gene expression profile plots for the three largest modules M1, M2, and M3. **(B)** Pathway overrepresentation analysis for the three largest modules M1, M2, and M3 using Reactome annotation.

**Fig. S2. qPCR validation of gene modulation in HCC cell lines**

**(A, B)** Validation of stable lentiviral PSD4 modulation by qPCR in **(A)** PLC/PRF/5 cells and **(B)** HepG2 cells. **(C, D)** Validation of stable lentiviral PSD4 modulation by qPCR in **(C)** PLC/PRF/5 cells and **(D)** HepG2 cells. **(E, F)** Validation of stable lentiviral CDC42 modulation by qPCR in **(E)** PLC/PRF/5 cells and **(F)** HepG2 cells. **(G, H)** Validation of stable lentiviral p65 modulation by qPCR in **(G)** HepG2 cells and **(H)** PLC/PRF/5 cells. Data represented as means ± SDs. **P*<0.05, ***P*<0.01 [one-way ANOVA].

**Fig. S3. ConTra-identified p65/RELA binding site on the PSD4 promoter**

ConTra v3 analysis of the PSD4 promoter. **(A, B)** p65/RELA binding site motifs (**(A)** M4444_1.02 and **(B)** MA0107.1) present on the PSD4 promoter. **(C)** Block 5 sequences of PSD4 promoter across primate species (chr2:113928995-113929333+; labelled 1-338) located approx. 2.2-2.6 kbp upstream of the PSD4 TSS (chr2:113931559+). The p65/RELA binding site motifs located approx. 2.5 kbp upstream of the TSS are highlighted in orange (M4444_1.02) and blue (MA0107.1).

**Fig. S4. MethPrimer-identified CpG islands on the PSD4 promoter sequence**

MethPrimer v2 analysis of 5 kbp sequence upstream of the PSD4 TSS reveals several CpG islands (blue highlights). Two islands are localized approx. 2.5 kbp upstream of the PSD4 TSS, proximate to the ConTra-identified p65/RELA binding site.

**Fig. S5. Construction and characterization of hepatocyte-specific PSD4-overexpressing transgenic mice**

**(A-C)** Generation of hepatocyte-specific PSD4-overexpressing transgenic mice. **(A)** M2 FLAG::Psd4 fusion construct subcloned into the murine *Alb* promoter-driven expression vector with a downstream SV40-polyA element. **(B)** M2-FLAG PCR screen in non-transgenic (non-TG) and transgenic (TG^Alb-PSD4^) mice (n=2 randomly-selected males (subjects 1 and 2), 1 female per cohort (subject 3)). **(C)** Immunoblotting confirming transgenic M2-FLAG::Psd4 overexpression in TG^Alb-PSD4^ hepatocytes (H) but not in non-TG hepatocytes (H) or TG^Alb-PSD4^ Kupffer (K) cells. Scant expression observed in Kupffer cell samples due to TG^Alb-PSD4^ hepatocyte contamination. **(D-F)** A test cohort of TG^Alb-PSD4^ mice (n=3 males, 3 females) and non-TG (n=3 males, 3 females) littermate controls were reared on a regular chow diet from birth to 250 days of age. **(D)** Growth rates, **(E)** liver weight/body weight ratios and **(F)** heart weight/body weight ratios of male and female PSD4 and non-TG littermates. Data represented as means ± SDs. **P*<0.05, ***P*<0.01 [one-way ANOVA].

**Fig. S1**


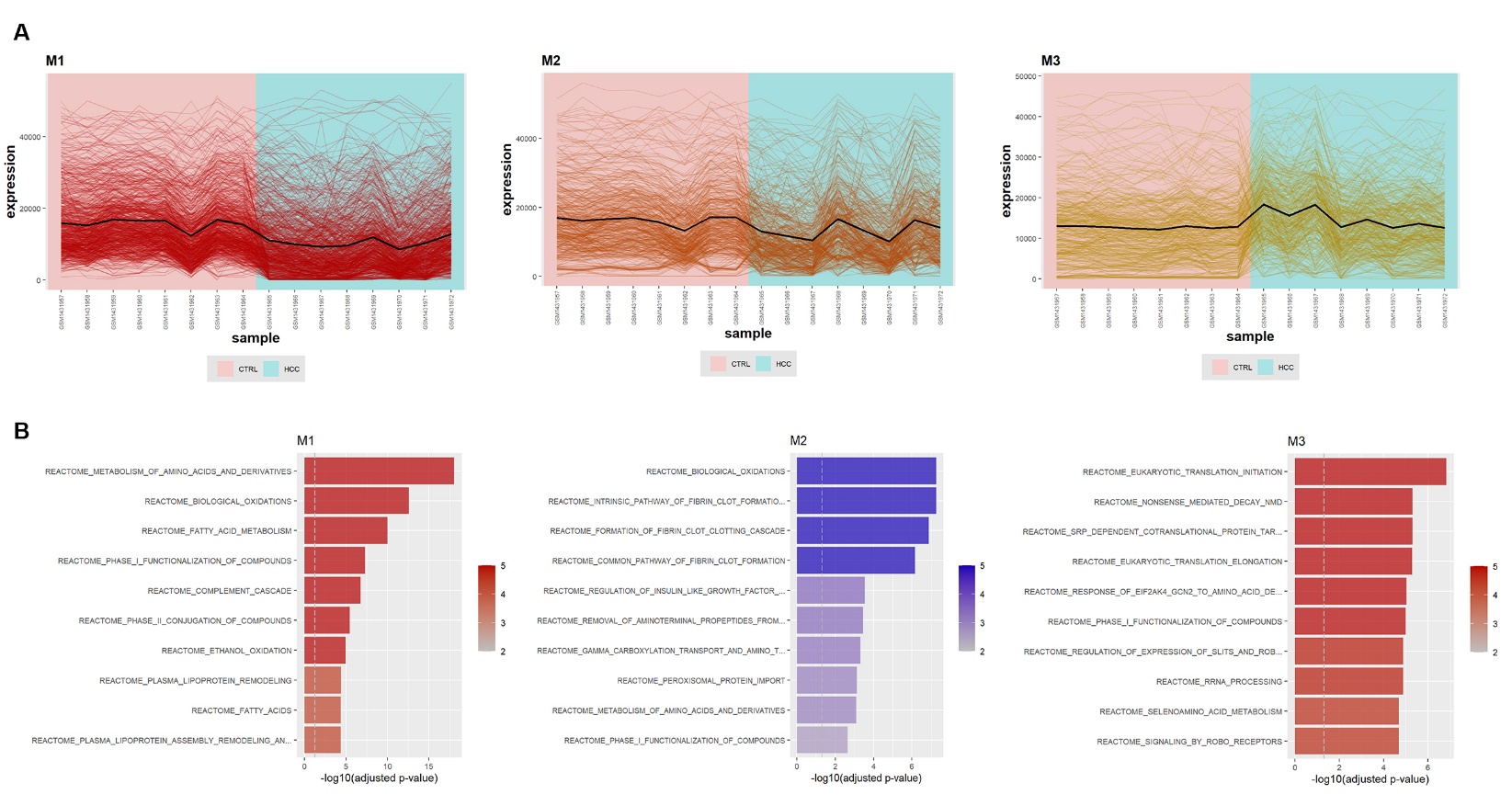


**Fig. S2**

**
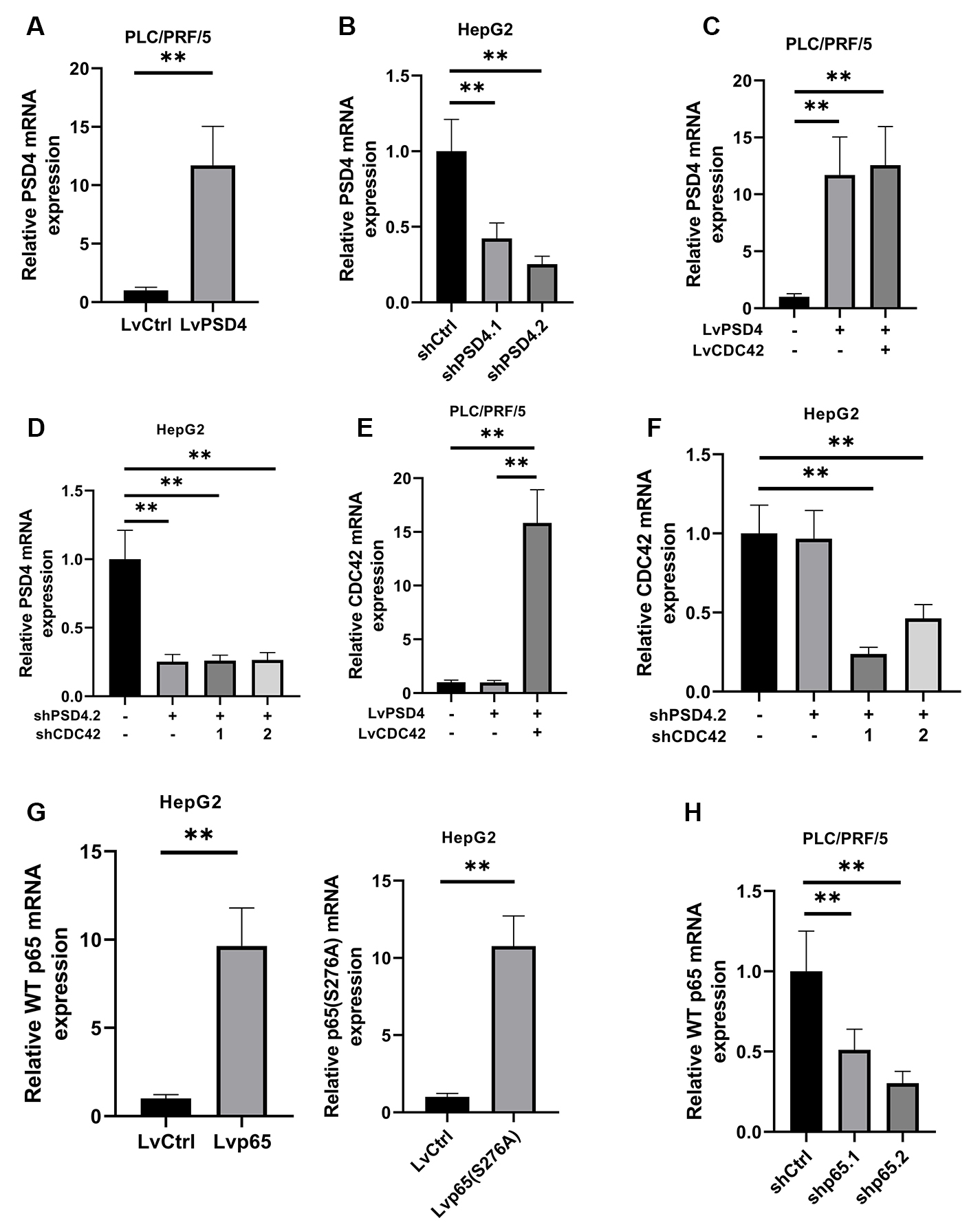
**

**Fig. S3**

**
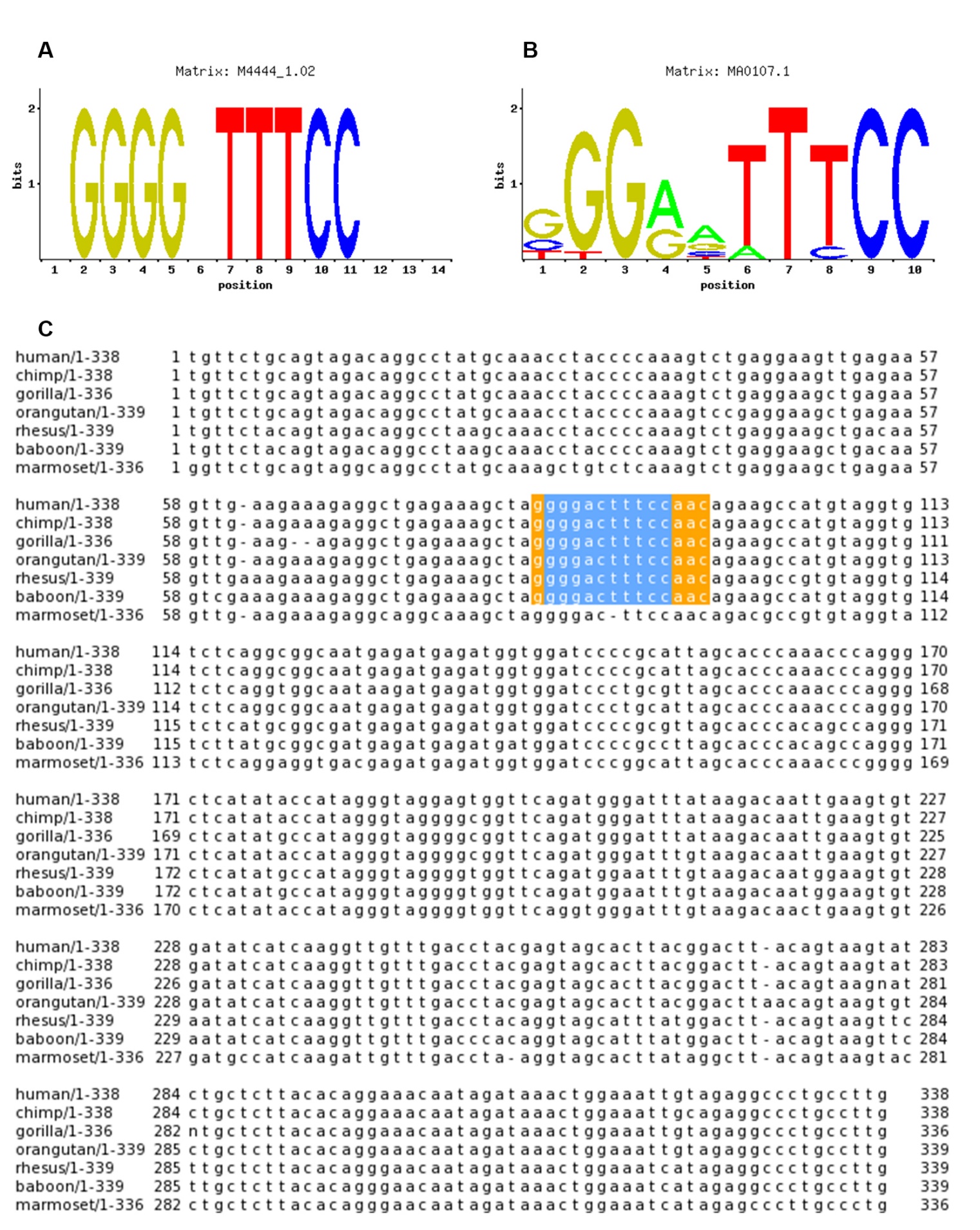
**

**Fig. S4**

**
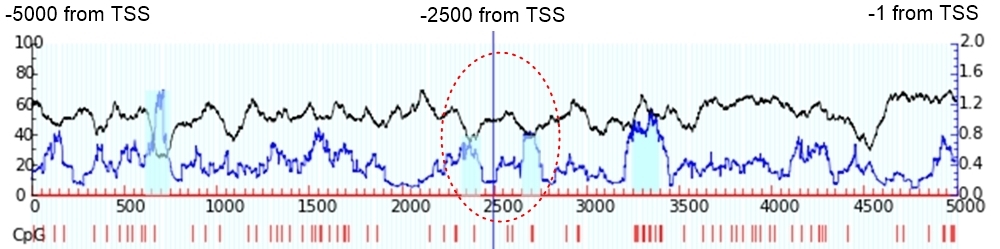
**

**Fig. S5**

**
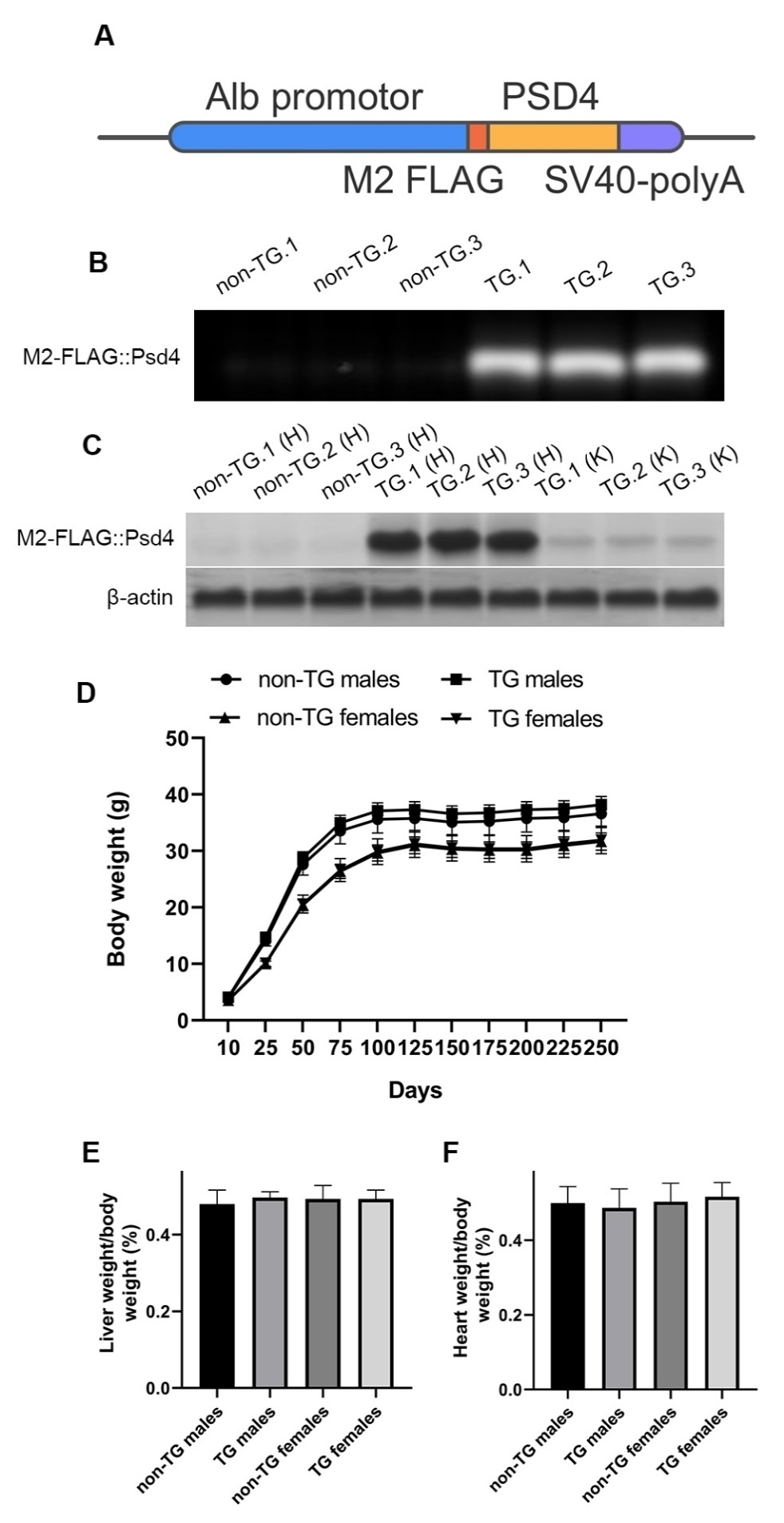
**

**REFERENCES FOR SUPPLEMENTARY INFORMATION**

[1] Udali S, Guarini P, Ruzzenente A, et al. DNA methylation and gene expression profiles show novel regulatory pathways in hepatocellular carcinoma. Clinical epigenetics. 2015; 7: 43.

[2] Russo PS, Ferreira GR, Cardozo LE, et al. CEMiTool: a Bioconductor package for performing comprehensive modular co-expression analyses. Bmc Bioinformatics. 2018; 19: 1-13.

[3] Nagji AS, Liu Y, Stelow EB, Stukenborg GJ, Jones DR. BRMS1 transcriptional repression correlates with CpG island methylation and advanced pathological stage in non‐small cell lung cancer. The Journal of pathology. 2010; 221: 229-37.

[4] Jiang H, Cao H-J, Ma N, et al. Chromatin remodeling factor ARID2 suppresses hepatocellular carcinoma metastasis via DNMT1-Snail axis. Proceedings of the National Academy of Sciences. 2020; 117: 4770-80.

[5] Zhou X, Xu M, Wang L, et al. Liver-specific NG37 overexpression leads to diet-dependent fatty liver disease accompanied by cardiac dysfunction. Genes & nutrition. 2016; 11: 14.

[6] Aparicio-Vergara M, Tencerova M, Morgantini C, Barreby E, Aouadi M. Isolation of Kupffer cells and hepatocytes from a single mouse liver. Alpha-1 Antitrypsin Deficiency: Springer, 2017; 161-71.
